# TLR3-Dependent Activation of TLR2 Endogenous Ligands via the MyD88 Signaling Pathway Augments the Innate Immune Response

**DOI:** 10.3390/cells9081910

**Published:** 2020-08-17

**Authors:** Hellen S. Teixeira, Jiawei Zhao, Ethan Kazmierski, Denis F. Kinane, Manjunatha R. Benakanakere

**Affiliations:** 1 Department of Orthodontics, School of Dental Medicine, University of Pennsylvania, Philadelphia, PA 19004, USA; hellen@upenn.edu (H.S.T.); kazmierskiethan@gmail.com (E.K.); 2 Department of Periodontics, School of Dental Medicine, University of Pennsylvania, Philadelphia, PA 19004, USA; jzha@med.wayne.edu; 3 Periodontology Department, Bern Dental School, University of Bern, 3012 Bern, Switzerland; dfkinane@outlook.com

**Keywords:** human gingival epithelial cells, TLR3, MyD88, HMGB1, Hsp60, pro-inflammatory cytokine

## Abstract

The role of the adaptor molecule MyD88 is thought to be independent of Toll-like receptor 3 (TLR3) signaling. In this report, we demonstrate a previously unknown role of MyD88 in TLR3 signaling in inducing endogenous ligands of TLR2 to elicit innate immune responses. Of the various TLR ligands examined, the TLR3-specific ligand polyinosinic:polycytidylic acid (poly I:C), significantly induced TNF production and the upregulation of other TLR transcripts, in particular, TLR2. Accordingly, TLR3 stimulation also led to a significant upregulation of endogenous TLR2 ligands mainly, HMGB1 and Hsp60. By contrast, the silencing of TLR3 significantly downregulated MyD88 and TLR2 gene expression and pro-inflammatory IL1β, TNF, and IL8 secretion. The silencing of MyD88 similarly led to the downregulation of TLR2, IL1β, TNF and IL8, thus suggesting MyD88 to somehow act downstream of TLR3. Corroborating in vitro data, Myd88^−/−^ knockout mice downregulated TNF, CXCL1; and phospho-p65 and phospho-IRF3 nuclear localization, upon poly I:C treatment in a mouse model of skin infection. Taken together, we identified a previously unknown role for MyD88 in the TLR3 signaling pathway, underlying the importance of TLRs and adapter protein interplay in modulating endogenous TLR ligands culminating in pro-inflammatory cytokine regulation.

## 1. Introduction

The molecular adaptor Myd88 participates in most Toll-like receptor’s (TLRs) inflammatory pathways. The role of Myd88 in the TLR3 pathway and the synergistic interaction of viral and bacterial recognition receptors has not been investigated. TLRs have an important role in the recognition of viral and bacterial pathogens and the subsequent activation of the innate immune responses in establishing homeostasis during an infection [1,2,3]. In homeostasis, an array of these pattern-recognition receptors (PRRs) of the immune system recognizes pathogen-associated molecular patterns (PAMPs) and instigates a proper immune response [1,4]. In dysbiosis, the overactivation of these TLRs may lead to chronic inflammation. The TLRs are identified by their localization (intracellular or extracellular) and corresponding ligands [5,6]. TLR1, TLR2, TLR4, TLR5, TLR6 and TLR11 are normally expressed on cell surfaces and detect microbial membrane components, such as lipoproteins and other microbial molecules. Conversely, TLR3, TLR7, TLR8 and TLR9 are intracellularly found in vesicles, such as endosomes, endoplasmic reticulum (ER), endolysosomes, and lysosomes, where they detect pathogen nucleic acid [1,7,8,9].

The oral mucosal surface is continuously exposed to commensals and pathogens due to the wide array of microorganisms present in the biofilm. The epithelium forms the first line of host defense against the invading pathogens [10,11,12,13]. In chronic oral infection, certain pathogens elicit unrestrained immune response affecting oral immune homeostasis. In periodontitis, human gingival epithelial cells (HGECs) play a crucial role in maintaining oral innate immune homeostasis by fending off bacterial attack by way of several mechanisms, but most notably through TLR activation [14]. The event is characterized by elevated pro-inflammatory cytokine and antimicrobial peptide production following bacterial perturbation. HGECs can express various TLRs, with TLR2 and TLR4 specifically recognizing the Gram − bacterial pathogen *Porphyromonas gingivalis* [15]. As such, TLR2 is regarded as an important receptor in sensing *P. gingivalis* and mediating innate immune responses [16]. TLR2 is involved in the detection of a wide range of pathogens derived from fungi, bacteria, viruses and parasites, including peptidoglycan and lipoteichoic acid from Gram + bacteria, lipoarabinomannan from *mycobacteria*, zymosan from fungi, hemagglutinin protein from the measles virus, tGPI-mucin (glycosylphosphatidylinositol-anchored mucin-like glycoproteins) from *Trypanosoma cruzi*, and lipopeptides from bacteria [17,18,19]. TLR2 generally forms heterodimers with TLR1 or TLR6 depending on the bacterial insult. The TLR1/2 heterodimer detects triacylated lipopeptides from mycoplasma and Gram − bacteria, whereas the TLR2/6 heterodimer recognizes diacylated lipopeptides from Gram + bacteria and mycoplasma [20,21,22]. Moreover, TLR2 works in cooperation with other co-receptors on the cell surface. Although it is believed that the TLR2 agonist mainly induces the production of inflammatory cytokines and not type I interferon by macrophages and dendritic cells, the production of type I interferon by inflammatory monocytes has been observed in response to vaccinia virus infection, which implies a cell type-specific role for TLR2 in antiviral responses [7,23,24,25]. While TLR2 is important in HGECs, we and others have shown that epithelial cells also express high levels of TLR3 compared to other TLRs [26,27]. In HGECs, we have shown that TLR3 can induce robust inflammatory cytokine responses through the activation of the mTOR signaling pathway [26]. TLR3 recognizes a synthetic double-stranded RNA (dsRNA) analog, polyinosinic:polycytidylic acid (poly(I:C)), and promotes the production of both inflammatory cytokines and type I interferon as an antiviral response [4,5,8,28,29], thus serving an important role in preventing virus infection [30,31,32].

The discovery of the Toll/interleukin-1 (TIR) domain on the adaptor molecule myeloid differentiation primary response 88 (MyD88) has led to intense studies of the TLR signaling pathways. The subsequent identification of additional TIR domain-containing adaptors has underscored the importance of the TIR domain in allowing TLRs to selectively recruit distinct adaptor molecules, thus providing specific immunological responses tailored to the infecting pathogen [33,34,35]. MyD88, the first identified member of this TIR family, is universally used by all TLRs, except TLR3, for the activation of the transcription factor NF-κB and mitogen-activated protein kinases (MAPKs) to stimulate inflammatory cytokines. By contrast, TIR-domain-containing adapter-inducing interferon-β (TRIF) is used by TLR3 and TLR4 and induces alternative pathways that result in the activation of transcription factors such as IRF3 and NF-κB, and the subsequent activation of type I interferon and inflammatory cytokines [36,37,38]. The current literature shows that Myd88 does not have a role in the TLR3 signaling network and that TLR3 is independent of Myd88 participation, however, the interaction of TLR3 with other TLRs and how this interaction influences MyD88-mediated proinflammatory gene expression has never been addressed. Having identified TLR3 as one of the important receptors in the HGEC’s inflammatory cytokine network, we hypothesized that its downstream signaling may control other TLRs’ expression and may participate in Myd88-dependent pro-inflammatory cytokine secretion via the expression of endogenous TLR ligands as observed in other systems [39,40,41].

## 2. Materials and Methods

### 2.1. Cell Challenge Assays

Primary human gingival epithelial cells (HGECs) were isolated from healthy young donors with the approval of the Institutional Review Board, from the University of Pennsylvania. HGECs were harvested at the 3rd passage, seeded at 0.5 × 10^5^ cells/well density in 6-well culture plates according our previously published method [12,26,42,43,44]. The cells were maintained in 2 mL of complete medium until 80% confluence and then washed twice with fresh medium and maintained in 2 mL of plain medium. At 90% confluence, the cells were introduced with a panel of bacterial and virus ligands (live *Porphyromonas gingivalis* ATCC strain 33277, heat-killed *P. gingivalis (P.g)*, Pam3CSK4, *P.g* LPS, poly I:C, *E. coli* K12 LPS, Flagellin from *Salmonella typhimurium,* FSL1 (Pam2CGDPKHPKSF), Imiquimod, and CpG oligonucleotides (ODN)) (all agonists were purchased from Invivogen, San Diego, CA, USA) for 24 h as previously described [45]. After 24 h of stimulation, the supernatant was collected, and TNF measurement was performed using enzyme-linked immunosorbent assay (ELISA) (R&D systems Inc., Minneapolis, MN, USA). After determining the ligands that preferentially upregulate proinflammatory cytokine, in the later experiments, cells were either stimulated with 1 µg/mL FSL-1 (TLR2/6 ligand), 5 µg/mL of poly I:C (TLR3 ligand) and 1 µg/mL Pam3CSK4 (TLR1/2 lingand), determined after the initial dose–response and agonist screening [26,45]. The culture supernatants were collected at the end of the experiment and stored at −80 °C until further use. The production of IL−8 and TNF was determined by ELISA according to the manufacturer’s instruction (R&D systems Inc., Minneapolis, MN, USA). None of the agonist stimuli affected cell viability, as determined by trypan blue exclusion, and as previously reported [26].

### 2.2. siRNA Assay

SMARTpool siTLR2, siTLR3 and MyD88 ON-TARGETplus SMARTpool and ON-TARGETplus non-targeting siRNA were from GE Healthcare Dharmacon, Inc., Lafayette, CO, USA. HGECs were transfected using GenMute™ siRNA Transfection Reagent for Primary Keratinocytes (SignaGen Laboratory, Rockville, MD, USA). Briefly, 100 nM final concentration of siRNA was used to transfect the cells at 50–60% confluence. Cells were stimulated with poly I:C 24 h post-transfection and harvested after another 24 h. The efficiency of knockdown was determined using mRNA and Western blot quantification, as reported in our published article.

### 2.3. Real-Time PCR

The cDNA was prepared by the cDNA archive kit (Thermo Fisher Scientific, Waltham, MA, USA) from the total RNA extracted from cultured cells using the RNeasy mini kit (Qiagen, Germantown, MD, USA). Real-time PCR was performed by the TaqMan technique of 50 ng of cDNA on the 7500 Fast system (Applied Biosystems, Waltham, CA, USA). TLR1, TLR2, TLR3, TLR6, IL-8, TNF, HMGB1, Hsp60, and GAPDH primers and probes were prepared as described previously [12]. The HGECs were grown to confluence and stimulated with Pam3CSK4 (1 µg/mL), FSL-1 (1 µg/mL), or poly I:C (5 µg/mL) for 30, 60, 90, 120 and 240 min and 4 and 24 h. The Quantitative TaqMan PCR-Array (Applied Biosystem) was performed based on our previously published microarray data on HGECs [26,46]. The fold increase was determined by the ΔΔCT method [47]. Mean log fold increase was used to derive the heatmap with two-way hierarchical clustering (rows = genes, columns = samples) using the MeV v4.1 software as previously described [26].

### 2.4. TNF and IL-8 Analysis by ELISA

The TNF and IL-8 levels in the supernatants of HGECs were determined by using the Quantikine ELISA kit (R&D Systems Inc.). All the experiments were performed in triplicate.

### 2.5. Animal Model

The animal study was conducted to test the hypothesis that the TLR3-dependent augmentation of pro-inflammatory cytokine production involves MyD88 in vivo. Experiments were performed using 8 adult female C57BL/6J mice (14–16 g) and 8 adult MyD88^−/−^ knockout mice (purchased from the Jackson Laboratory, Bar Harbor, ME, USA) per group, with a protocol approved by the University of Pennsylvania Institutional Animal Care and Use Committee. The rostral back of the animals was trimmed with an electric shaver and subsequently removed. Poly I:C was topically applied on mice for 6, and 12 h. A chromophore containing 25 and 8 mg of poly I:C was applied on the shaved skin of the back (2.5 cm × 2 cm); the best time point and concentration were selected (8 mg for 12 h) based on the preliminary findings (data not shown). Control mice were treated similarly with a vehicle chromophore [2,19,48,49]. Animals were euthanized under isoflurane sedation, and their skin was acutely dissected [18]. Skin was fixed in 4% paraformaldehyde, frozen in an optimal cutting temperature (OCT) compound (Tissue-Tek, Sakura Finetek, Torrance, CA, USA), cut in 8 μm sections on a cryostat, and stored at −80 °C until further use. The slides were defrosted the next day and fixed in 10% formaldehyde for 10 min. After an appropriate period, the slides were immersed in methanol at −20 °C for 5 min, and then washed in PBS three times, for 5 min each. The samples were blocked in 10% horse serum for 30 min at room temperature, and again washed in 1× PBS. Primary antibodies (p-IRF3 (S386), P-NF-KappaB p65 (S536), Isotype and TNF alpha from Cell Signaling Technologies, MA, USA) were added and the reaction mixture was incubated in a humid chamber overnight at 4 °C (primary antibodies were diluted 1:800 and 1:100 times, according to the manufacturer’s instructions, using 0.5% horse serum [49,50]. On the next day, the samples were incubated with a secondary antibody (Alexa Fluor from Cell Signaling Technology, Danvers, MA, USA) in a humid chamber for 1 h at room temperature. Secondary antibodies were diluted 1:100 or 1:200 (concentration used) times using 0.5% horse serum). The slides were mounted with a cover slip using Prolong^®^ Gold anti-fade reagent with DAPI and mounting media (P-36931, Thermo Fisher Scientific), and stored for 20 min in a dark drawer. The edges were sealed with nail vernix and slides were observed under a microscope [48].

All the images for each specific antibody were captured using the same condition. Images captured from 3 to 4 skin sections obtained from each animal were imaged at a magnification of 60×. The intensity of immunofluorescence within the epidermis was measured using ImageJ v1.8.0_112 (subtracting background fluorescence), and the mean immunofluorescence was determined for each mouse (8 mice per group) [51,52,53,54].

### 2.6. Statistical Analysis

Statistical analysis was performed by Prism 6.0 (GraphPad, La Jolla, CA, USA). Data were analyzed with one-way ANOVA followed by Tukey’s multiple comparison tests. 

## 3. Results

### 3.1. TLR3 Stimulation Leads to Robust TLR2 Transcriptional Activation

**Figure 1:**
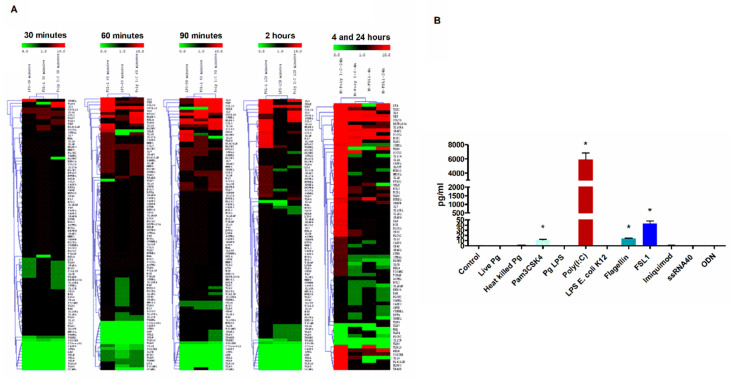
TLR3 stimulation of human gingival epithelial cells (HGECs) leads to robust activation proinflammatory signaling: the cells were treated with various TLR ligands for 24 h and the supernatant was subjected to TNF measurement using ELISA. TLR3 stimulation by poly I:C induced robust TNF cytokine induction (**A**). HGECs were incubated with *E. Coli* LPS (1 µg/mL), FSL-1 (1 µg/mL) and poly I:C (5 µg/mL) for 30, 60, 90, 120 min and 4 and 24 h. Total RNA was isolated, converted to cDNA and customized qPCR-Arrays were analyzed. The ΔΔCT values were used to generate the heatmaps based on two-way hierarchical clustering with MeV v4.1 software (rows = genes, columns = samples) using the values from two independent experiments. The color scale indicates relative expression: Red, above mean; green, below mean; and black, below background (**B**). Statistical comparison was done for ELISA by one-way ANOVA followed by Tukey’s multiple comparison test (* *p* < 0.05) and results are represented as mean ± SE (*n* = 3) from three independent experiments.

We have previously shown that TLR3 activation in HGECs induces robust pro-inflammatory response via the mTOR signaling network [26]. Here, we further investigated if other TLR signaling pathways could contribute to this robustness in pro-inflammatory network activation in HGECs. Similar to the previously examined time-dependence of treatment [12,14,26,42,55], the early and late activation of the inflammatory response was investigated (30, 60, 90 and 120 min and 4 and 24 h) after stimulation. We observed that poly I:C induced higher TLR2, TLR4 and TLR7 gene expression compared to FSL-1 and LPS at earlier time points. In comparison with other ligands, poly I:C induced a robust activation of pro-inflammatory genes (Figure 1A). Surprisingly, the cells stimulated with poly I:C for 24 h induced significantly higher TLR2 levels than the TLR2 specific ligand (Figure 1A).

**Figure 2:**
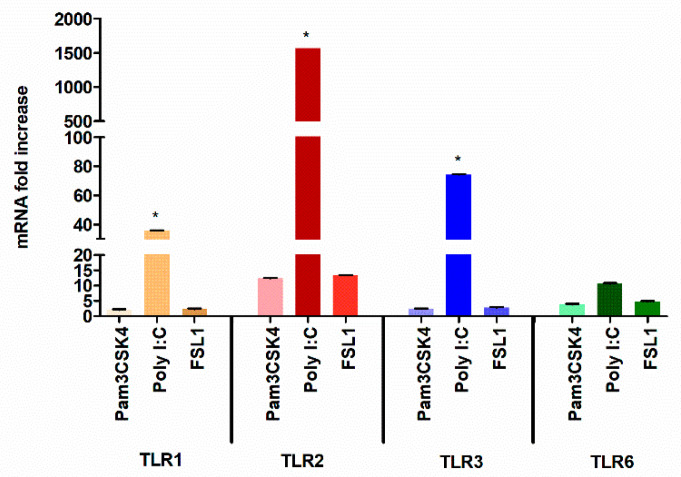
TLR3 stimulation induced high levels of TLR2 gene expression: HGECs were treated Pam3CSK4 (1 µg/mL), FSL-1 (1 µg/mL) and poly I:C (5 µg/mL) for 4 and 24 h. Quantitative real-time PCR was performed on cDNA as stated above. Poly I:C induced higher TLR3 gene expression at 4 h, but at 24 h post stimulation, TLR3 activation induced significantly higher TLR2 gene expression. Statistical test: one-way ANOVA followed by Tukey’s multiple comparison test (* *p* < 0.05). Results are mean ± SE (*n* = 3) from three independent experiments.

Furthermore, to confirm these findings, HGECs were treated with a panel of TLR ligands and examined for TNF production at 24 h. Our data shows that poly I:C induced higher TNF production compared to other TLR ligands (Figure 1B), which is in agreement with our previous observation [26]. It is well known that TLR2 can heterodimerize with TLR1 or TLR6 to induce Myd88-mediated signaling [10,11,12,13,14,56,57,58]. To determine if there is any difference in the levels of TLR1, TLR2 and TLR6 expression, the HGECs were treated with Pam3CSK4 (TLR1/2), FSL-1 (TLR2/6) and poly I:C (TLR3) ligands for 24 h and examined by real-time PCR. Unexpectedly, the poly I:C induced higher TLR2 expression levels than TLR3 (Figure 2). Since we observed significantly higher TLR2 gene expression and TNF production by poly I:C stimulation at 24 h, we hypothesized that robust pro-inflammatory mediators produced by TLR3 signaling was partly through the activation of TLR2.

### 3.2. TLR3-Mediated Expression of TLR2 Requires MyD88 Signaling

**Figure 3:**
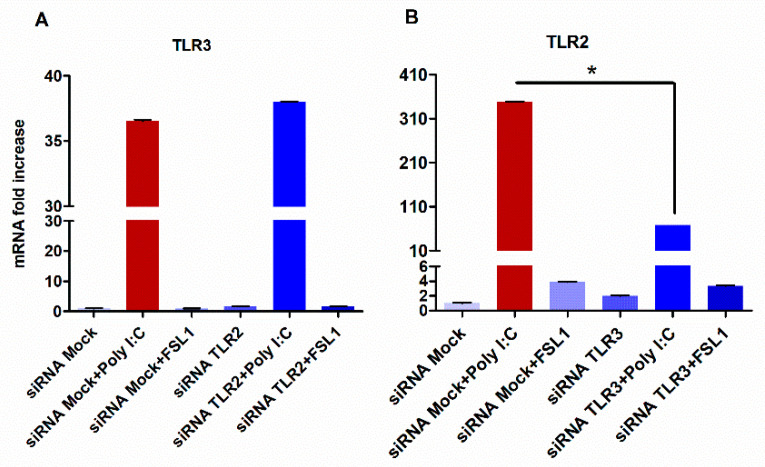
Silencing of the TLR3 reduced TLR2 gene expression in HGECs: when TLR2 was silenced, the expression of TLR3 was not affected after stimulation with poly I:C (**A**). On the other hand, silencing TLR3 downregulated TLR2 gene expression (**B**). Statistical test: one-way ANOVA followed by Tukey’s multiple comparison test (* *p* < 0.05) and the results are represented as the mean ± SE (*n* = 3) from three independent experiments.

To test whether TLR3 can impact the expression levels of TLR2, TLR3 was silenced by siRNA and the then HGECs were stimulated with poly I:C. As shown in Figure 3B, the silencing of TLR3 led to the significant downregulation of TLR2. Conversely, the silencing of TLR2 and subsequent stimulation with FSL1 produced no effect on TLR3 expression levels (Figure 3A).

**Figure 4:**
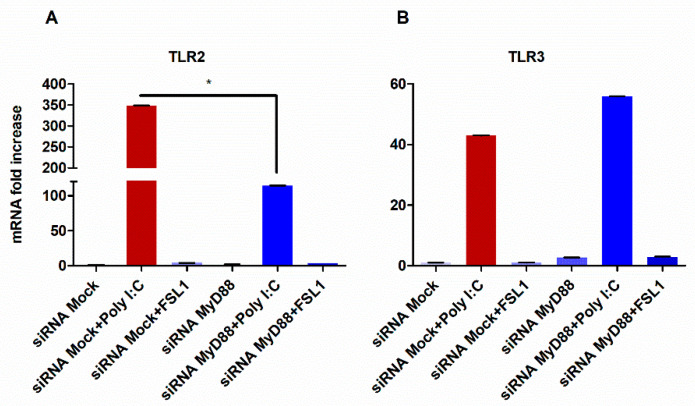
Upregulation of TLR2 by poly I:C is partly mediated by MyD88: when MyD88 is silenced, the expression of TLR2 is significantly decreased after the stimulation with poly I:C. (**A**). However, the silencing of Myd88 did not alter the expression of TLR3 (**B**). Statistical test: one-way ANOVA followed by Tukey’s multiple comparison test (* *p* < 0.05) and the results are represented as the mean ± SE (*n* = 3) from three independent experiments.

It is well documented that TLR2 signaling requires MyD88 adapter molecule and that TLR3 does not require MyD88 to induce downstream signaling [10,58,59,60]. Therefore, we investigated whether the ability of TLR3 to impact TLR2 expression was mediated through MyD88. To accomplish this, MyD88 was silenced by siRNA and the HGECs were subsequently stimulated with poly I:C for 24 h. Accordingly, the silencing of MyD88 downregulated TLR2 expression levels, while no change in the TLR3 levels was observed (Figure 4A,B). Together, our data demonstrate the requirement of MyD88 in TLR3-mediated TLR2 expression.

### 3.3. TLR3 Signaling Potentiate Pro-Inflammatory Cytokine Secretion Partially through the Activation of TLR2 and MyD88

**Figure 5:**
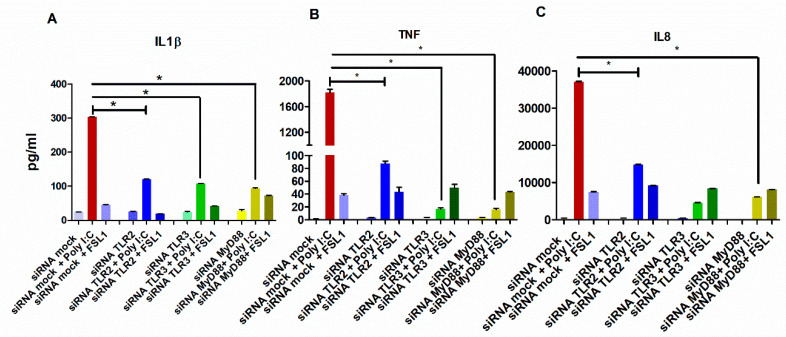
Poly I:C-upregulated cytokine production is partially through the activation of TLR2 and MyD88. The HGECs were stimulated with poly I:C and FSL1 for 24 h after silencing TLR2, TLR3 and MyD88. ELISA on the supernatant showed a significant decrease in IL1β (**A**), TNF (**B**) and IL8 (**C**) levels. Moreover, TLR2 silencing significantly downregulated IL1β, TNF and IL8 secretion upon poly I:C treatment. The striking difference was observed when MyD88 was silenced, where IL-1β, TNF and IL8 secretion was significantly downregulated upon poly I:C treatment. These data underline the unexpected role of MyD88 in the MyD88-independent pathway. Statistical test: one-way ANOVA followed by Tukey’s multiple comparison test (* *p* < 0.05) and the results are represented as the mean ± SE (*n* = 3) from three independent experiments.

To provide insight into the role of MyD88, we investigated the differential expression profiles of TLR2, TLR3, and MyD88 after silencing with siRNAs and the subsequent exposure to their respective ligands. First, we examined the silencing of TLR2. As expected, TLR2 expression levels were minimal in the absence and presence of its ligand, FSL-1 (Figure 5A). Interestingly, stimulation with the TLR3 ligand, poly I:C, could restore the expression levels of TLR2 and do so more robustly than with FSL-1 alone (Figure 5A). Additionally, the silencing of TLR3 and subsequent exposure to poly I:C resulted in the increased expression of MyD88 by two-fold changes. (Figure 5C).

**Figure 6:**
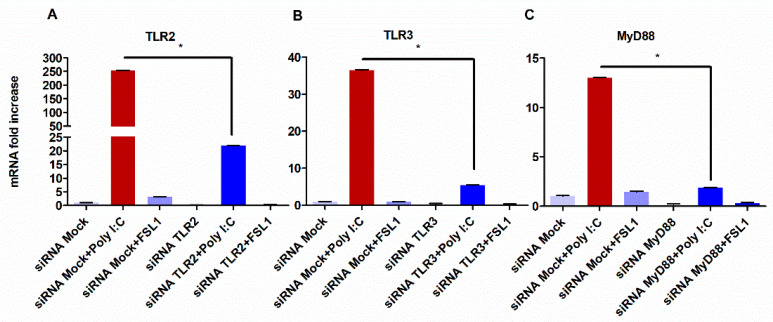
TLR3 stimulation induced MyD88 and inhibition attenuated its expression: the HGECs were treated with respective ligands after silencing TLR2, TLR3 and MyD88. Silencing TLR3 induced significantly higher TLR2 expression even when TLR2 was silenced (**A**). As expected, siTLR3 treated the cells’ downregulated TLR3 (**B**). Interestingly, poly I:C-treated cells significantly increased Myd88 expression (**C**). However, when MyD88 was silenced, poly I:C significantly downregulated MyD88 expression. The extent of poly I:C increasing TLR2 expression was relatively higher than that of FSL-1 increasing TLR3 expression. Statistical test: one-way ANOVA followed by Tukey’s multiple comparison test (* *p* < 0.05) and the results are represented as the mean ± SE (*n* = 3) from three independent experiments.

Then, we sought into whether the downregulation of TLR2 or MyD88 could have an impact on cytokine secretion. To accomplish this, the TNF and IL8 levels were quantified from HGECs silenced by TLR2 and after subsequent stimulation with poly I:C. As shown in Figure 6A,B, both the TNF and IL8 levels were significantly reduced. Similarly, the poly I:C treatment of silenced MyD88 HGECs showed a significant reduction of TNF and IL8 levels, an observation in line with the expected interaction between TLR2 and MyD88.

### 3.4. Activation of TLR2 by TLR3 is Mediated via the Induction of Endogenous Ligands HMGB1 and Hsp60

**Figure 7:**
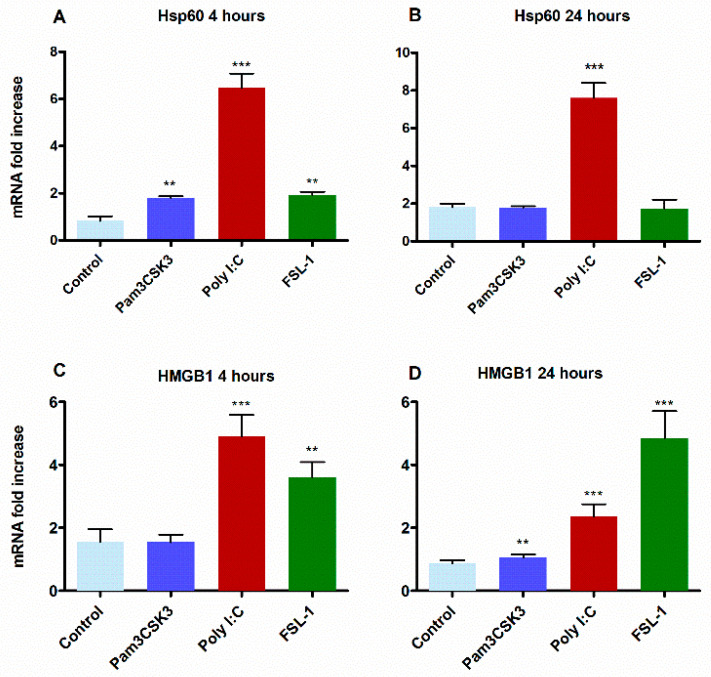
TLR3 stimulation activated endogenous TLR2 ligands: the HGECs were stimulated with Pam3CSK3, poly I:C and FSL-1 for 4 and 24 h. The cDNA was used to measure HMGB1 and Hsp60 gene expression. Poly I:C increased the expression of both the Hsp60 (HSPD1) and HMGB1 genes. Poly I:C increased the significantly higher HMGB1 expression at the 4 h time point (**A**), whereas FSL1 induced higher the HMGB1 at 24 h (**B**). On the other hand, Hsp60 expression was minimally activated by FSL-1 treatment but robustly upregulated by poly I:C at the 4 and 24 h time points (**C**,**D**). Statistical test: one-way ANOVA followed by Tukey’s multiple comparison test (* *p* < 0.05, ** *p* < 0.01, *** *p* < 0.001) and the results are represented as the mean ± SE (*n* = 3) from three independent experiments.

Recent investigations into microbial molecules and their signaling pathway in host cells have identified several host-derived endogenous ligands [61,62]. These endogenous molecules include proteins that activate TLR signaling during pathological processes even in the absence of microbial PAMPs [63,64,65,66]. These ligands comprise various types of molecules, such as proteins, fibronectin, heparin sulphate, biglycan, fibrinogen, oligosaccharides, and nucleic acids [62,67,68,69,70,71]. Key proteins include high-mobility group box 1 (HMGB1), heat shock proteins (HSP), tenascin-C, cardiac myosin, and S100 proteins [64,66,71], of which HMGB1 and Hsp60 have been specifically shown to activate the TLR2 signaling network [62,72,73]. To better understand the role of TLR2 in TLR3-mediated transcriptional activation, HGECs were stimulated with poly I:C and the TLR2 agonists Pam3CSK3 and FSL-1 for 4 and 24 h and examined for HMGB1 and Hsp60 expression levels. Stimulation by poly I:C produced maximum levels of HMGB1 after 4 h (Figure 7A), while FSL-1 induced maximum levels of HMG1 after 24 h (Figure 7B). Additionally, poly I:C robustly promoted the expression levels of Hsp60 at both time points, while a minimal effect was observed for stimulation with FSL-1 (Figure 7C,D).

**Figure 8:**
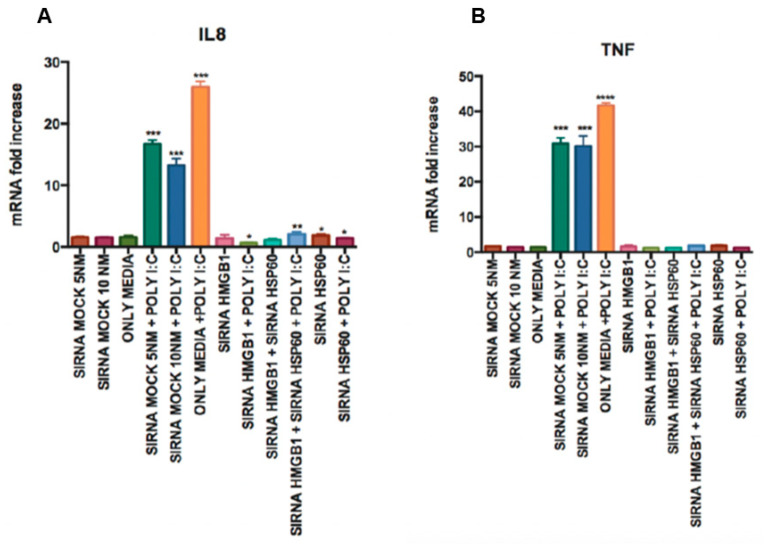
Silencing of HMGB1 and Hsp60 leads to the downregulation of pro-inflammatory cytokines: IL8 (**A**) and TNF (**B**) were downregulated upon poly I:C stimulation, and the silencing of HMGB1 and Hsp60 in epithelial cells. Statistical test: one-way ANOVA followed by Tukey’s multiple comparison test (* *p* < 0.05, ** *p* < 0.01, *** *p* < 0.001, **** *p* < 0.0001) and the results are represented as the mean ± SE (*n* = 3) from three independent experiments.

Then, the TNF and IL-8 levels were measured to check the functionality of HMGB1 and HSP60 when they were silenced. When we treated poly I:C in Hsp60 and HMGB1-silenced HGECs, the TNF and IL-8 expression levels were significantly downregulated in comparison to the levels observed with cells treated with poly I:C and siRNA mock. This shows the role of HMGB1 and HSP60 in the functional activation of TLR2 in inducting TNF and IL-8 expression (Figure 8A,B).

### 3.5. Myd88^−/−^ Knockout Mouse Downregulated the Pro-Inflammatory Genes and Transcription Factors NF-κB and IRF3

**Figure 9:**
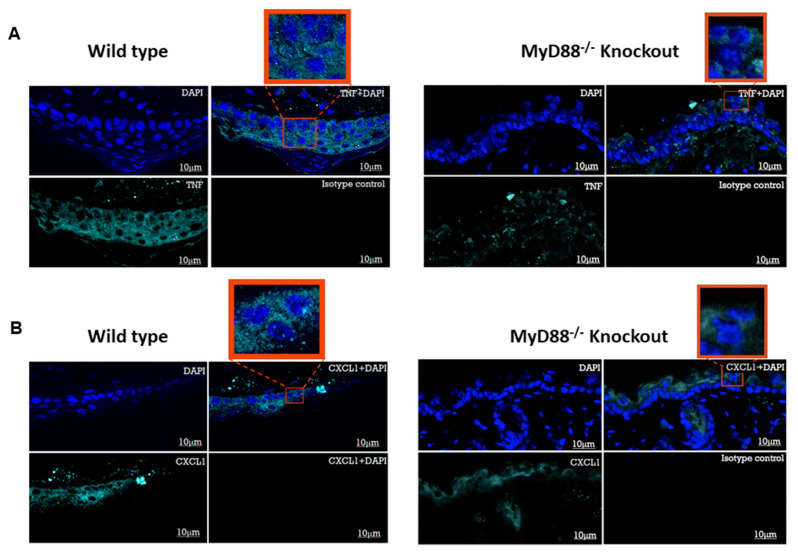
MyD88 knockout mice display a blunted proinflammatory response to poly I:C stimulation: the skins of wild-type mice and MyD88 knockout mice were challenged with poly I:C for a given time point and immunofluorescence imaging was performed on skin tissue sections. A higher mean fluorescence intensity was observed on the skin surface of the wild-type mouse (green (TNF); blue (DAPI)). (**A**). Similarly, a higher mean fluorescence intensity was observed on the skin surface of the wild-type mouse (green (CXCL1); blue (DAPI)) (**B**). (N = 8 mice per group in total (two independent experiments with *n* = 4 animals/group). Bar = 10 μm).

In vitro findings prompted us to confirm the results using Myd88^−/−^ knockout mice. We adopted a well-known mouse model of skin infection to look at the expression of pro-inflammatory genes TNF and CXCL1 and the transcriptional factors NF-κB and IRF3 after the treatment with poly I:C or the vehicle. Interestingly, we noticed a majority of immuno-positive cells on the epithelial layer of wild-type mice compared to the Myd88^−/−^ group treated with poly I:C. We also encountered few immuno-positive cells on the connective tissue which could be due to the paracrine effect of TNF or CXCL1 and is expected as the connective tissue have fibroblasts and immune cells which also express TLR3 in their endosome organelles. Wild-type mice showed significantly higher TNF positive cells compared to the Myd88^−/−^ mice group (Figure 9A). Similarly, wild-type mice showed significantly higher CXCL1 positive cells compared to the Myd88^−/−^ group (Figure 9B).

**Figure 10:**
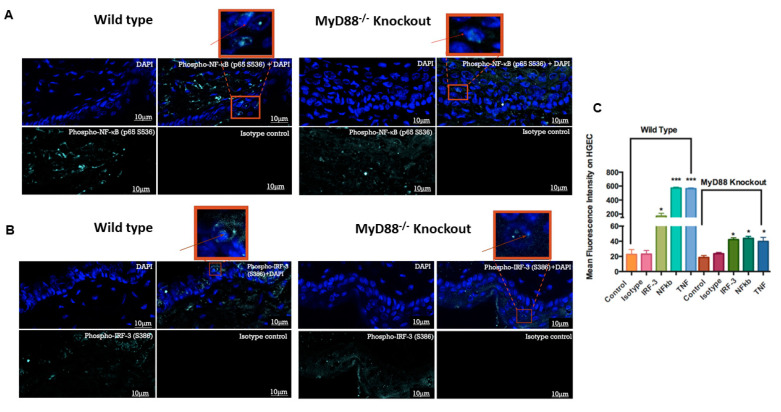
MyD88 knockout mice display downmodulated transcriptional factors to poly I:C stimulation: the skin of wild-type mice and MyD88 knockout mice challenged with poly I:C and immunofluorescence performed using phospho-NF-kB p65 (S536) antibody in skin tissue sections. A higher mean of fluorescence intensity was observed on the surface skin of the wild-type mouse (green (phospho-NF-kB p65); blue (DAPI)) (**A**). Furthermore, the skin tissue was stained for phospho-IRF3 (S386). A higher mean of fluorescence intensity was observed on the surface skin of the wild-type mouse (green (phospho-IRF3); blue (DAPI)) (**B**). (N = 8 mice per group in total (two independent experiments with *n* = 4 animals/group). Bar = 10 μm)). The intensity of immunofluorescence within the epidermis was quantified using ImageJ software. The mean fluorescence intensity was higher in the stained cells of the wild-type mouse compared to the values for the MyD88^−/^^−^ knockout mice (**C**). Statistical test: we performed one-way ANOVA, followed by Tukey’s multiple comparison test (* *p* < 0.05). The results represent the mean ± SE values from 8 animals per group in total (two independent experiments with *n* = 4 animals/group).

Furthermore, we used phospho-antibodies against NF-kB and IRF3 to determine the effect of activated transcription factors that might have facilitated the significantly higher expression of TNF and CXCL1 in the wild-type mice. Phospho-NF-kB in a wild-type group showed significantly higher positive cells compared to the Myd88^−/−^ mice group (Figure 10A). Similarly, phospho-IRF3 in wild-type group showed significantly higher positive staining compared to the Myd88^−/−^ knockout group (Figure 10B). The statistical differences in the mean immunofluorescence intensity values for phospho-NF-κB, phospho-IRF3, TNF and CXCL1 were significantly higher in the wild-type mice challenged with poly I:C, as compared to the values for the Myd88^−/−^ knockout group challenged with poly I:C (Figure 10C). These data show that Myd88^−/−^ is somehow involved in eliciting the immune response triggered by TLR3 stimulation.

## 4. Discussion

The binding of ligands to TLRs induces downstream signaling via two distinct pathways, i.e., the MyD88-dependent and TRIF-dependent pathways, for the induction of pro-inflammatory cytokines and (interferon) IFN genes [74,75]. MyD88 is used by TLR2, TLR5, TLR7, TLR8 and TLR9, while TRIF is used by TLR3. TLR4 uses both MyD88- and TRIF-adaptor molecules [37,76]. However, the involvement of TLR3 with the MyD88 signaling network remains controversial. While Takumi, Kawasaki et al., 2014, advocates that the TLR3 signaling pathway is totally independent of the MyD88 adaptor protein [77], a study by Xia et al. [50] indicates that both TLR3- and MyD88-dependent signaling play important roles in shaping the development of humoral responses to the single-cycle vaccine RepliVax WN (West Nile virus vaccine) [50]. Our study findings concur with Xia et al., 2013, showing the poly I:C-mediated upregulation of TLR2 levels in siTLR2-treated cells; this suggests that TLR3 stimulation leads to not only a strong transcriptional activation of TLR2, but also functionally potentiates innate immune response. Furthermore, our data show that poly I:C causes an increase in MyD88 expression, even though it is thought to be independent of the TLR3 signaling network.

**Figure 11:**
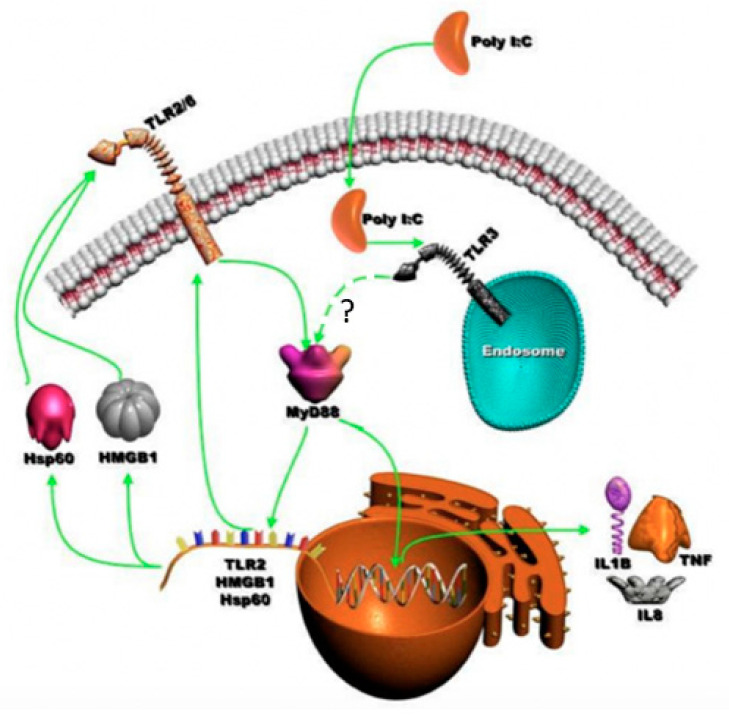
Schematic diagram of the TLR3-dependent activation of MyD88 signaling: TLR3 activation triggers endogenous ligands of TLR2 (HMGB1 and Hsp60) via the usage of the MyD88 signaling pathway. These endogenous TLR2 ligands then stimulate TLR2 signaling to augment the innate immune responses.

The recognition of pathogens by epithelial cells involves interplay between PAMPs and various host PRRs. The occurrence of this interaction during infection results in the release of various inflammatory mediators and chemokines that induce an influx of neutrophils into the site of infection, activating nearby macrophage- and antigen-presenting cells, and ultimately decides the acquired immune response [78]. Bacterial LPS has been shown to upregulate TLR3 expression via the TLR4–MyD88–IRAK–TRAF6–NF-κB-dependent signaling pathway, for enhancing anti-viral responses [78,79]. However, the role of TLR3 in bacterial infections is poorly understood and remains controversial [9,24,65]. Although TLR3 is identified as a major MyD88-independent PRR for the induction of type-1 IFN, in response to different viral infections, the role of TLR3 in bacterial infections is poorly understood [80]. Nevertheless, the direct interaction between TLR3 and the MyD88 TIR domain needs to be fully evaluated (Figure 11) especially due to the findings that ligand binding to TLR3 homodimer brings the TIR domain in proximity of the MyD88 TIR domain poised for activation [81]. Moreover, our data demonstrate the interactions of MyD88 in the regulation of TLR2 expression by TLR3, which suggests that Myd88 transcriptional activation is indirect and may augment proinflammatory cytokine production by epithelial cells. In addition, we for the first time showed that the upregulation of TLR2 occurred in poly I:C-treated cells, as TLR2 silencing significantly downregulated IL-8 and TNF secretion. When MyD88 was silenced, the secretion of both TNF and IL-8 was significantly downregulated upon poly I:C treatment. These data emphasize an unexpected role of MyD88 in the MyD88-independent signaling pathway. Additionally, we elucidated the role of HSMGB1 and HSP60 in the downstream activation of TLR2 by a TLR3-specific ligand. This perhaps results in the generation of robust inflammatory signaling of TLR2 that might lead to persistent inflammation. In other words, the presence of viral ligands may potentiate bacterial recognition receptors to become overactive and induce elevated proinflammatory cytokines.

In a seminal paper, Lai et al. investigated a skin injury model (full-thickness incisions were performed creating a circular wound) of mice and identified the fact that inflammation was driven by TLR3 mediated responses in keratinocytes [28,59,82]. The results showed the necessity of TLR3 for the induction of inflammation after a skin injury. Interestingly, staphylococcal lipoteichoic acid (LTA)-mediated TLR2 signaling suppressed TLR3 signaling via the induction of the negative regulatory factor TRAF1 [8,83,84,85]. The authors observed that the RNA from necrotic epithelial cells triggered TLR3 in undamaged epithelial cells, leading to the release of pro-inflammatory cytokines, and concluded that the specificity of a ligand and its response is dictated by a cell type-specific TLR2 ligand [49,73,85]. In our experiment, the HGECs behaved differently than expected. TLR3 ligand stimulation in HGECs led to the synergistic activation of TLR3 and TLR2 via the induction of the endogenous ligand for TLR2. In our study, when TLR3 is triggered by poly I:C, TLR2 is upregulated, although in siTLR2 treated cells, suggesting that TLR3 stimulation leads to a robust transcriptional activation of TLR2. Furthermore, TLR3 stimulation via poly I:C induced the endogenous ligands of TLR2 in vitro. In addition, poly I:C increased MyD88 expression even though it was thought to be independent. Complementing the in vitro data, TNF and CXCL1 secretion were significantly downregulated upon poly I:C treatment in MyD88-deficient mice. Taken together, the above data suggest that TLR2–TLR3 regulatory networks are complex and reveal their association with the TLR3–MyD88–TLR2 signaling axis. However, we cannot rule out the possibility of other signaling pathways, as it is also possible that other signaling pathways may tandemly participate in the transcriptional activation of TLR2 and/or MyD88, mediating enhanced cytokine production. We are aware of previous studies claiming that TLR3 signaling is independent of MyD88. Based on our data, we cannot specifically claim the direct evidence for the TLR3–MyD88 physical interaction. Nonetheless, we cannot rule out an indirect involvement of MyD88 in TLR3 signaling. Further studies are needed to confirm the direct role for MyD88 in the TLR3 signaling network.

Interestingly, viral infections occurred in the fetal membranes that secreted MIP-1β and RANTES [79], in response to poly I:C, via MyD88 signaling [32,33,35]. This suggests their association with the TLR3–MyD88 signaling, which probably depends on the cell type specificity and type of stimulus. Nonetheless, there are no reports suggesting the involvement and cooperation of MyD88 in TLR3 signaling pathways in oral keratinocytes. In this pathway, TLR3-activated TLR2 expression via a Myd88-dependent mechanism, and TLR3 mediated the upregulation of endogenous ligands for TLR2, mainly HMGB1 and Hsp60. We believe that this discovery regarding the co-operation among TLRs for the activation might result in persistent inflammation if a viral infection has occurred. On the contrary, studies have provided evidence regarding the protection provided by the TLR3 and MyD88-dependent signaling pathways against viral infections. The increased susceptibility and high mortality of viral infections has been noted in MyD88^−/−^ and TLR3^−/−^ knockout mice at the early stage of infection [8,50]. These data corroborate a comprehensive examination of the roles of these pathways interacting with each other during the development of long-term adaptive immune responses to viruses. The impact of the lack of TLR3 or MyD88-dependent signaling is also manifested during B cell memory development [50]. When MyD88 knockout mice were treated with RepliVAX WN to determine the response of B cells, a significant reduction in B cell activation was observed. This study also noted that the activation of TLR2 occurred via TLR3 in a downstream direction, through HSP60 [50]. Our data on MyD88^−/−^ corroborates with these observations where poly I:C stimulation downregulated p65 (S536) and pIRF3 (S386), leading to the downregulation of CXCL1 and TNF when compared to wild-type mice. Although these observations may point out an evidence for MyD88 involvement in TLR3 signaling, we recognize that the data presented here are not enough to claim the direct interaction of TLR3–MyD88. There may be several other molecules independently triggering TLR signaling pathways to induce cytokine response in the absence of MyD88. Hence, MyD88 may not be a central player in the TLR3 signaling network but may play a bystander role.

The human oral cavity (mouth) hosts a complex microbiome, consisting of bacteria, archaea, protozoa, fungi and viruses. These bacteria are responsible for the two most common diseases of the oral cavity: periodontal (gum) disease and dental caries (tooth decay) [86]. The focus has traditionally been on bacteria when discussing the microbiological aspects of oral disease. However, poly infections including viruses are probably more involved in the diseases associated with the oral cavity than has been previously thought. The role of several viruses in ulceration is well known, but viruses of the herpes family may play a role in periodontal disease, and papillomaviruses have been implicated in oral cancer [87]. It should be noted that microbial activity can also induce viral replication, as has been shown recently in the case of (Epstein-Barr Virus (EBV) and malaria. If the impact of viral replication on the bacterial environment is real, then it might be expected that the bacterial profiles would differ between the sites, with or without viruses. Such correlations have been previously reported [88].

Epithelial cells in the mucosa serve as a barrier for those micro-organisms and recognize them through pathogen recognition receptors (PRRs) that instigate antibacterial and antiviral responses. The mechanism of co-operation between TLRs may help explain the complexity of viral and bacterial infections. The results of the current study indicate that both TLR3- and MyD88-dependent signaling play an important role in shaping the development of innate immune responses and may help explain the complications of bacterial and viral infections in chronic oral inflammation. On the beneficial side, we believe that poly I:C could be used as an excellent choice as an adjuvant in the development of vaccines against chronic oral infections such as periodontitis to mount robust immune responses [89].
